# Emulated muscle spindle and spiking afferents validates VLSI neuromorphic hardware as a testbed for sensorimotor function and disease

**DOI:** 10.3389/fncom.2014.00141

**Published:** 2014-12-04

**Authors:** Chuanxin M. Niu, Sirish K. Nandyala, Terence D. Sanger

**Affiliations:** ^1^Department of Rehabilitation, Ruijin Hospital, School of Medicine, Shanghai Jiao Tong UniversityShanghai, China; ^2^Department of Biomedical Engineering, University of Southern CaliforniaLos Angeles, CA, USA; ^3^Biokinesiology, University of Southern CaliforniaLos Angeles, CA, USA; ^4^Neurology, University of Southern CaliforniaLos Angeles, CA, USA

**Keywords:** spindle, motor control, afferents, emulation, neuromorphic

## Abstract

The lack of multi-scale empirical measurements (e.g., recording simultaneously from neurons, muscles, whole body, etc.) complicates understanding of sensorimotor function in humans. This is particularly true for the understanding of development during childhood, which requires evaluation of measurements over many years. We have developed a synthetic platform for emulating multi-scale activity of the vertebrate sensorimotor system. Our design benefits from Very Large Scale Integrated-circuit (VLSI) technology to provide considerable scalability and high-speed, as much as 365× faster than real-time. An essential component of our design is the proprioceptive sensor, or muscle spindle. Here we demonstrate an accurate and extremely fast emulation of a muscle spindle and its spiking afferents, which are computationally expensive but fundamental for reflex functions. We implemented a well-known rate-based model of the spindle (Mileusnic et al., 2006) and a simplified spiking sensory neuron model using the Izhikevich approximation to the Hodgkin–Huxley model. The resulting behavior of our afferent sensory system is qualitatively compatible with classic cat soleus recording (Crowe and Matthews, 1964b; Matthews, 1964, 1972). Our results suggest that this simplified structure of the spindle and afferent neuron is sufficient to produce physiologically-realistic behavior. The VLSI technology allows us to accelerate this behavior beyond 365× real-time. Our goal is to use this testbed for predicting years of disease progression with only a few days of emulation. This is the first hardware emulation of the spindle afferent system, and it may have application not only for emulation of human health and disease, but also for the construction of compliant neuromorphic robotic systems.

## Introduction

The multi-scale nature of human nervous system makes it difficult to measure all relevant information about sensorimotor function. Moreover, such inadequacy of multi-scale information also prevents us from understanding the mechanism of neurological diseases, whose expression depends on networks and interconnectivity of neurons. In the case of movement disorders in childhood, the ultimate clinical effect of cellular injury may take years or even decades to fully emerge (Sanger, 2003) due to a complex interplay among the child's injury, development, and experience. Therefore, the lack of detailed and complete sensorimotor measurements, both in multi-scale and across long timespan, makes it extremely difficult to quantify the mechanism of disease or to predict the efficacy of long-term treatments.

Here, we attempt to understand sensorimotor functions using a synthetic approach. This requires creating sensorimotor models that are biologically realistic but emulated on non-biological hardware. The history of synthetic approaches in neuroscience dates back to the 1940s, when scientists started creating artificial neurons and neural networks using electronic circuits (McCulloch and Pitts, 1943). Models of neuron dynamics (Hodgkin and Huxley, 1952; Rosenblatt, 1958) soon emerged to be simulated on digital computers. Since the 1980s, special-purpose hardware with Very Large Scale Integrated-circuit (VLSI) technology started to benefit from some of the key insights in neural computation, including asynchrony among neurons, spike representation of information, and self-improving mechanisms such as plasticity (Mead, 1989; Serrano-Gotarredona et al., 2009; Indiveri et al., 2011; Neftci et al., 2014). This category of designs, termed “neuromorphic” hardware, has been successful in understanding mechanisms of memory (Chicca et al., 2003), visual representation (Lichtsteiner et al., 2008), and recently cognitive function (Eliasmith et al., 2012). For sensorimotor function, the synthetic approach should not only describe neurons, but also the physiological environment (muscles, proprioceptors, peripheral nerves, skeletal system) that the neurons interact with. If the goal is to predict long-term changes of sensorimotor function, the emulation speed must exceed real-time. These requirements pose a major challenge when designing the hardware, as the running speed of may decrease significantly with model complexity. In this case, our approach for sensorimotor modeling must accommodate enough detail for multi-scale simulation, but it must also maintain high speed when scaling to larger network sizes.

Field Programmable Gate Array (FPGA), a programmable VLSI device, can parallelize neuron simulations at very high speed (Guerrero-Rivera et al., 2006; Li et al., 2010). The inherent parallel structure of FPGAs permits that when model complexity increases, the running speed can be maintained by adding units. The parallel design uses each clock cycle to update multiple neurons, access a wide range of memory, and send out neuron spikes for inter-chip communication; therefore FPGAs are able to accomplish more calculations than clock-cycled computers at the same central clock frequency. In our previous work (Niu et al., 2012), we showed the technical details of the emulation platform using FPGAs, which achieved a running speed 365× faster than real-time for a simulation of the spinal components of the monosynaptic stretch reflex. In the current study, we simulate the muscle spindle proprioceptors and their attached sensory afferent neurons. The spindle system is an essential part of the monosynaptic stretch reflex, as well as a major source of state information for feedback control and stabilization. Because of the model complexity of spindles, there has not previously been an attempt to simulate them in hardware. Therefore, our high-speed hardware simulation of the muscle spindle will provide an essential element for studying the stretch reflex as well as for understanding stabilization and feedback control of biological and neuromorphic motor systems.

Among the many studies investigating muscle spindles in mammals (Eldred et al., 1962; Lennerstrand, 1968; Boyd et al., 1977; Hulliger, 1984), the series of experiments conducted by Matthews and colleagues (Crowe and Matthews, 1964b; Matthews, 1964, 1972) characterized cat soleus spindles in considerable detail. Their representative data provide the reference for the rate-based spindle model (Mileusnic et al., 2006) chosen for our study. We expect our spike-based emulation to enrich the original rate-based spindle model: all spiking afferents will be available for analysis, and the spiking behavior should be compatible with physiological recording. The advantage of converting to a spike-based representation is that we can compare our results directly against physiologically-measured signals, we can investigate questions related to the number or firing pattern of sensory afferents, and we can investigate the effect of spike timing on plasticity. For the purpose of emulating sensorimotor function and disease, we argue that the model of spindle afferents does not have to include all anatomical details, but its outcome must satisfy at least two constraints: (1) distinguishable firing pattern between Group Ia and Group II afferents, and the difference should be compatible with physiological data; (2) distinguishable change in behavior that reflects changes in dynamic and static gamma fusimotor drive. We verified the VLSI emulation against these two constraints. Because of the physiological differences between cats and humans (Prochazka and Hulliger, 1983), we also directly compared our emulated signals with human spindle afferents (Edin and Vallbo, 1990) to see whether this model can be used to emulate human data. If successful, our results will provide a testbed with sufficient detail to represent known sensorimotor physiology in order to describe and predict the effect of neural injuries over many years.

## Materials and methods

### Apparatus

**Figure 1:**
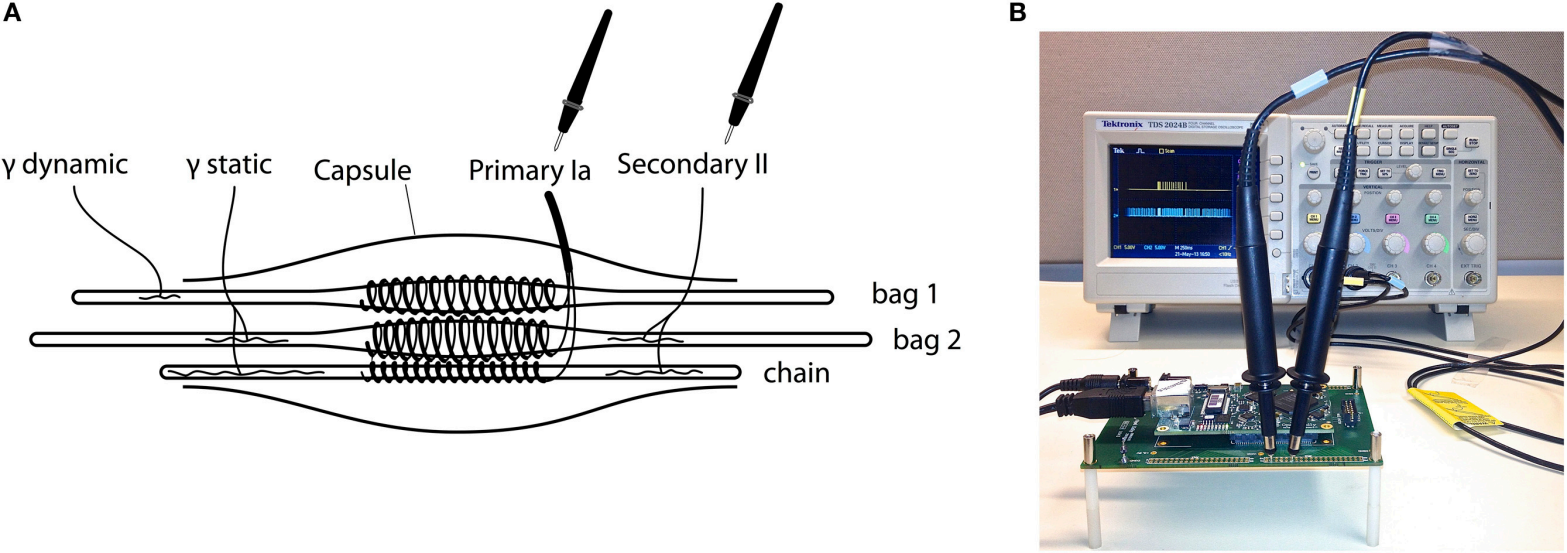
**(A)** Illustration of the proprioceptive sensory structures emulated in this study. A detailed spindle model (Mileusnic et al., 2006) included three types of fibers (bag1, bag2, and chain), two groups of afferents (Primary Ia and Secondary II) responding to two types of fusimotor drives (gamma dynamic and static). The firing rate produced by the spindle model was converted to excitatory post-synaptic currents (EPSC) and directly activated a total of 256 sensory neurons, 128 for Group Ia and 128 for Group II. **(B)** The spiking behavior of a single afferent neuron is monitored using “virtual neuron recording” by attaching oscilloscope probes to the output pins from the FPGA. The emulation was slowed down to real-time such that spikes were spaced out on the display. The virtual stretching of muscle was programmed on-chip, but could be replaced by any muscle length measured from synthetic muscles, robot joints, cadaveric specimens, etc.

The emulated neurological system comprises a muscle spindle with three types of fibers (bag1, bag2, and chain), two groups of afferents (Group Ia and II) responding to two types of fusimotor drives (gamma dynamic and static), illustrated in Figure 1A. The mathematical descriptions of muscle spindle are implemented using (FPGA, Xilinx Spartan-6), a programmable version of VLSI electronic chips.

We favor FPGAs over pipelined hardware such as Graphic Processing Units (GPUs) or clustered CPUs due to its inherent parallelism that resembles neural circuitry. It is also argued that when networking multiple units for large-scale disease emulation, FPGAs are significantly more flexible for enabling custom-built communication protocols, including neuromorphic transmission protocols that directly transmit neuron-like spikes.

### FPGA hardware design

The implementation requires translating the equations of neurons and spindles into digital circuits described in Verilog Hardware Description Language (HDL). We enforce a combinatorial circuit design as opposed to a sequential clocked design when translating all models, therefore we can maximize performance since circuits do not have to wait for clock signal boundaries. A central scheduler periodically updates all emulated models and distributes the latest information to corresponding parts. Each update generates neurological activities accounting for 1 ms in the real world. The frequency of update can be easily adjusted on digital VLSI, therefore when increasing the updating frequency the emulation will operate faster than real-time. The speed of emulation can keep increasing as long as all models finish their state update within each accelerated clock cycle. The maximal speed of emulation is constrained by how fast the electronic signals can propagate through the combinatorial circuits.

Figure 1B shows a working setup of the hardware platform. The spiking activities of up to 80 neurons are measurable directly from the FPGA using an oscilloscope for “virtual neuron recording,” which on-site verifies the accuracy of emulation with hardware in the loop. Other activities are transferred to a data-logging computer for offline analysis. Note that when collecting data for offline analysis, the emulation has to be slowed below real-time due to limited bandwidth between the FPGA and the datalogging computer; the emulation is accelerated to 365× real-time only when investigating long-term changes. The FPGA communicates with the data-logging computer through OpalKelly development kits (XEM6010, OpalKelly Inc.). More technical details can be found in Niu et al. (2012).

#### Floating-point arithmetics in combinational logic

Spindle models are evaluated in IEEE-754 single-precision floating-point numbers. Typical floating-point arithmetic IP cores are either pipe-lined or based on iterative algorithms such as CORDIC, all of which require clocks to schedule the calculation. In our testbed, no clock is allowed for model evaluation thus all arithmetics need to be executed in pure combinational logic. The implementations of adder and multiplier are inspired by the open source project “Free Floating-Point Madness,” available at http://www.pldworld.com/_hdl/2/_ip/www.hmc.edu/chips/fpmul.html. The modified code used in this study is available upon request.

Floating-point division is more resource demanding than multiplications. We approximated the division with additions and multiplications therefore avoided the direct implementation of floating-point division. Our approach is inspired by an algorithm described by Lomont (2003), which provides a good approximation of the inverse square root for any positive number *x* within one Newton-Raphson iteration:

(1) $$Q\left(x\right)=\frac{1}{\sqrt{x}}\approx x\left(1.5-\frac{x}{2}\cdot x^{2}\right)\left(\text{x}>0\right)$$

where Q(x) only contains additions and multiplications. Any division with a positive divisor can be achieved if two blocks of Q(x) are concatenated:

(2) $$\frac{a}{b}=\frac{a}{\sqrt{b}\cdot \sqrt{b}}=a\cdot Q\left(b\right)\cdot Q\left(b\right)\left(\text{b}>0\right)$$

It is trivial to adjust this algorithm for negative divisors (b < 0).

#### Serialize neuron evaluation using time-shared multiplexing

Consider that the spindle model only needs to update at 365 kHz even in the highest speed (1 ms time granularity, 365× real-time), there is room for time-sharing the FPGA logic-gates among neurons, thus using fewer logic-gates to emulate larger amount of neurons. The maximal number of neurons that can be serialized (*N*_*serial*_) is constrained by the following relationship:

(3) $$C\times N_{serial}\times 365\times F_{emu}\leq F_{fpga}$$

Here *F*_*fpga*_ is the fastest clock rate that a FPGA can operate on; *C* is the minimal clock cycles needed for updating each state variable in the on-chip memory, in our case *C* = 2 due to an optimized design for memory access; *F*_*emu*_ = 1 kHz is the time granularity of emulation (1 ms), and 365× F_*emu*_ represents 365× real-time. Consider that Xilinx Spartan-6 FPGA devices have a maximum 200 MHz central clock frequency, the theoretical maximum of neurons that can be serialized is

(4) $$N_{serial}\leq \frac{200MHz}{2\times 365\times 1kHz}\approx 274$$

In the current design we choose *N*_*serial*_ = 128.

### Emulation of muscle spindles

As the sensory organ that provides the main source of proprioceptive information, a typical muscle spindle produces afferents Group Ia (transducing muscle length and a function of velocity) and Group II (primarily sensing muscle length). The activity of spindle is modulated by two types of fusimotor drive (gamma dynamic and gamma static). At present there are no analytical equations that can accurately describe the dynamics of a spindle. However, spindle behavior can be approximated using ordinary differential equations for numerical solutions. Though few such models are available, the one presented by Mileusnic et al. (2006) showed a close fit to the firing rate recorded from cat soleus spindles. We implemented this spindle model as the first step to deduce the essential elements for realistic spindle afferents.

The chosen spindle model used differential equations to describe the relationship between afferent firing rates and the instantaneous muscle status (length and lengthening velocity). The overall structure of the spindle model can be summarized as follows:

(5) $$\;\dot{R}\left(t\right)=f\left(L,\dot{L},\gamma_{dynamic},\gamma_{static},t\right)$$

(6) $$R\left(t\right)=\int \dot{R}\left(t\right)dt\;$$

where *R*(*t*) is the instantaneous firing rate of an afferent fiber at time *t*, *L* is the length of the muscle, γ_*dynamic*_ and γ_*static*_ are the firing rates of gamma fusimotor drives. This model described each spindle fiber as coupled springs and damping; it characterized how three types of fiber (bag1, bag2, nuclear chain) contribute to each group of spindle afferent (Ia and II). The thixotropic property of spindle, i.e., that the spindle output depends on the history of stretching (Hasan and Houk, 1975), was not captured in this model.

A full combinatorial implementation of the spindle model requires more silicon resources than available on a single Spartan-6 FPGA chip. Therefore, we optimized the model by trimming its transcendental functions and replacing some on-chip calculations with pre-calculated values. For example, a subset of the model for bag1 fiber is shown below:

(7) $${\dot{x}}_{0}=\left(\frac{\Gamma_{dynamic}}{\Gamma_{dynamic}+\Omega^{2}}-x_{0}\right)/\tau \;$$

(8) $${\dot{x}}_{1}=x_{2}\;$$

(9) $${\dot{x}}_{2}=\frac{1}{M}\left[T_{SR}-T_{B}-T_{PT}-\Gamma_{1}x_{0}\right]$$

where

(10) $$T_{SR}=K_{SR}\left(L-x_{1}-L_{SR0}\right)\;$$

(11) $$T_{SR}=\left(B_{0}+B_{1}x_{0}\right)\cdot \left(x_{1}-R\right)\cdot CSS\cdot {\left|x_{2}\right|}^{0.3}$$

(12) $$T_{PR}=K_{P}R\left(x_{1}-L_{P}R0\right)\;$$

refer to Mileusnic et al. (2006) for the definitions of variables. As can be seen from Equation (11), the absolute value of lengthening velocity (*x*_2_) was raised to the power of 0.3, which is trivial to program in Matlab/C++ but has no corresponding syntax in Verilog. We approximated |*x*_2_|^0.3^ as |*x*_2_|^0.25^, because the power of 0.25 is straightforward to acquire by iterating on *Q*(*x*) of Equation (1):

(13) $$Q\left(Q\left(x\right)\right)={\left(\left(x^{-0.5}\right)\right)}^{-0.5}=x^{0.25}\left(x>0\right)$$

In addition, most of the equations were expanded to polynomials, such that the constant parts in the equations were pre-calculated without consuming multipliers, e.g., 1/*M* in Equation (9) was pre-calculated as a single number instead of doing an on-chip division.

After optimization we are able to host an entire spindle using only 60% of a single Spartan-6 FPGA chip, with a running speed peaking at 500× real-time. Typically a single Spartan-6 chip can support one spindle connected with at least 1024 neurons at 365× faster than real-time.

### Emulation of spiking neurons

From the perspective of information theory, the function of a neuron is to convert post-synaptic currents into a train of binary spikes with limited bandwidth (Sanger, 2011). In an emulated neurological system focusing on its functional role, a neuron can be modeled to any level of detail as long as it satisfies the protocol of “current-in, spike-out.” In the current study we adopted the neuron model developed by Izhikevich (2003), which approximates the Hodgkin–Huxley model (Hodgkin and Huxley, 1952). In our emulation, we set the four parameters (a, b, c, d) required in the Izhikevich model to ensure all sensory neurons fit Hodgkin's description of Class 1 excitatory neurons (Hodgkin, 1948; Izhikevich, 2007). Class 1 excitatory neurons are one of the major types found in human spinal cord recordings (Prescott et al., 2008). Since the firing rate of Class 1 excitatory neurons is a monotonic representation of post-synaptic current over a large dynamic range, it allows straightforward conversion from the spindle output (in firing rate) to the input of the neuron (in post-synaptic current).

Although we enforce a pure combinatorial design to maximize the running speed, neurons still time-share physical circuits in order to maximize the population size with limited silicon surface. Pseudorandom white uniform noise (5 mV amplitude) was added to the membrane potential of each neuron to introduce variability for population firing. The noise is introduced to represent the large number of inputs that a neuron usually receives from the dendritic tree. The noise level was set to create a typical 4.8 mV fluctuation in the membrane potential (Fellous et al., 2003). Pseudorandom noise is generated using a linear-feedback-shift-register (George and Alfke, 2001).

### Spiking responses of spindle to virtual muscle stretch

There has been a long-lasting history studying rapid excitatory responses of a muscle following stretch that dates back to 1751 by Robert Whytt (Pearce, 1997). Over the past centuries it has been revealed that the process of response is a complex muscle reaction, with multiple excitatory responses occurring at different latencies following a muscle stretch (Hammond, 1955). We emulated a series of classic muscle stretch experiments that were originally performed in cat soleus muscles (Crowe and Matthews, 1964b; Matthews, 1964, 1972). Since these datasets are matched in the original spindle model (Mileusnic et al., 2006) to produce compatible firing rates, our emulation is intended to produce compatible spike patterns under similar muscle stretch. Four types of stretch stimulus were introduced to the emulated spindle: linear stretch, tap, sinusoidal stretch and release. We focus on whether the spindle afferents show: (1) distinguishable firing pattern between Group Ia and Group II afferents, and the difference should be compatible with physiological data; (2) distinguishable change in behavior that reflects changes in dynamic and static gamma fusimotor drive.

We further analyzed the emulated spindle activity using a white-noise approach, which has been widely used in system identification for non-linear biological systems (Marmarelis, 2004). We stimulated the emulated spindle using low-pass filtered white noise. It is expected that the firing rate reconstructed from our emulation should be statistically correlated with the firing rate produced by the rate-based spindle model. In addition, the emulated spindle activity was compared to spindle afferent recordings from humans. Due to the known difference between spindles of cats and humans (Prochazka and Hulliger, 1983), we do not expect our emulation to exactly match human data but the comparison should reflect the known difference.

It is noteworthy that all virtual recordings in this study required slow emulation below real-time, otherwise the amount of data produced by FPGA per unit time would exceed the bandwidth for data logging. The mathematical correctness of emulation when accelerated to the full 365× real-time speed is ensured by in-loop fault checks.

## Results

### Qualitative difference between Ia and II afferent spikes

**Figure 2:**
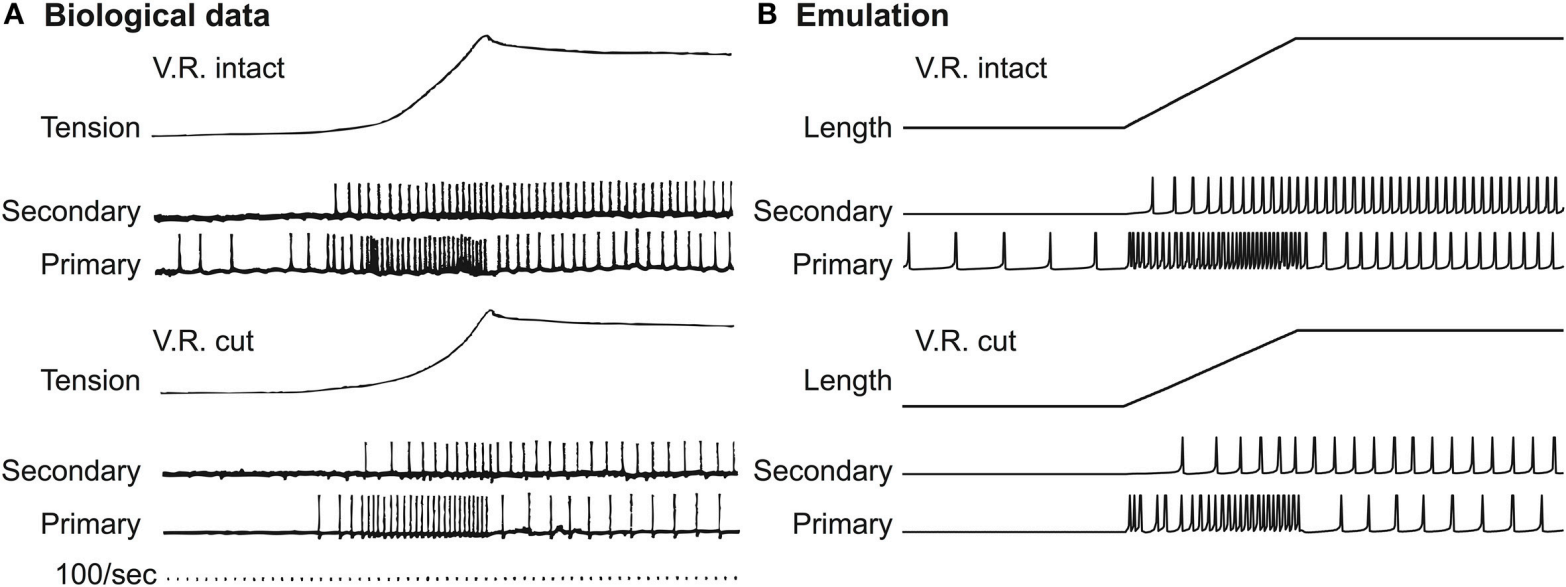
**Afferent spiking in response to stretch-and-hold stimulation. (A)** Cat data. In cat soleus spindles there is a clear differentiation between primary and secondary afferent fibers when responding to stretch stimuli (Matthews, 1972), reproduced with permission. **(B)** FPGA emulation. The action potential of emulated sensory neurons show clearly distinguishable patterns between Primary and Secondary fibers, similar to the spike patterns observed in cat recording. Note that muscle tensions were originally shown in **(A)** not for demonstrating the stimuli; the actual waveform of stimuli was similar to the Length curve shown in **(B)**.

We first replicated the stretch-and-hold experiment (Matthews, 1972) conducted with the soleus muscle of a decerebrate cat. In their work, the cat soleus muscle was stretched by 14 mm within 200 ms with the muscle maintained elongated after stretching. The spiking responses of cat were measured using intramuscular recording and are shown in Figure 2A (reproduced with permission). Responses were recorded both in the presence (ventral roots intact) and absence (ventral roots cut) of tonic fusimotor activity. In both cases the Primary (Ia) afferent exhibited stronger phasic response than Secondary (II) afferent, especially when the stretching velocity is greater than zero. In contrast, the Secondary (II) afferent produced stronger tonic response during the entire process of stretching.

Equivalent stretch-and-hold experiments were tested in VLSI emulation. Note that the original spindle model requires all muscle lengths normalized to the length of relaxed muscle (resting length, *L*_0_). The spindle was stretched to 36.8% of *L*_0_, corresponding to the 14 mm elongation of cat soleus muscle (Matthews, 1972) from an average rest length of 38 mm (Scott et al., 1996). Figure 2B shows the emulated action potentials in response to the 0.368*L*s_0_ virtual stretch lasting for 200 ms. We observed a clear distinction between Ia and II afferents in the emulated results. It can also be seen that in the presence of gamma fusimotor drive (ventral roots intact), both Ia and II afferents were more active in a similar way between real and emulated responses. The difference between Ia and II afferents is qualitatively similar to physiological data.

Note that in Figure 2A the muscle tensions were presented to show the muscle's mechanical response to external stretch, whereas the actual waveform of stretch should be in the shape shown in Figure 2B (muscle length). In our emulation, the muscle tension was only implicitly calculated for the spindle as an intermediate variable, and was hence not directly measurable.

### Qualitative difference between static and dynamic fusimotor drive

**Figure 3:**
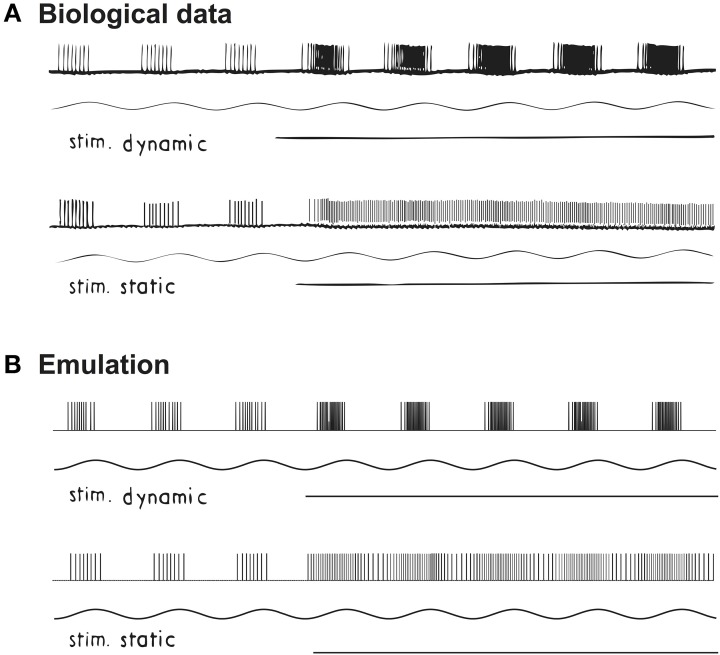
**Changes in proprioceptive responses to fusimotor drives under sinusoidal stretch. (A)** Spikes recorded from a primary ending of cat soleus muscle when stretched at 3 Hz with peak-to-peak 1 mm. Reproduced from Crowe and Matthews (1964a) with permission. **(B)** Emulated spindle producing similar Primary (Ia) and Secondary (II) afferent spikes. The main effect of gamma dynamic stimulation is to increase the phasic response in spindle afferent neurons; while gamma static mainly increases the tonic response, as expected.

We also compared the biological and emulated afferents from a primary afferent neuron, when the spindle fiber is stimulated with different fusimotor drives. In particular, two fusimotor drives (gamma dynamic and static) were selectively stimulated during a sinusoidal stretch of 1 mm peak-to-peak at 3 Hz (Crowe and Matthews, 1964a). The original recording is shown in Figure 3A. External stimuli to the gamma fibers were activated between the 3rd and 4th cycle (horizontal line in Figure 3A). As expected, the effect of gamma dynamic drive was to facilitate the phasic response of cat soleus spindle, while the tonic responses were mostly facilitated by gamma static drive. Emulated spindle afferent spiking is shown in Figure 3B. A virtual sinusoidal stretch of 0.026*L*_0_ peak-to-peak at 3 Hz was applied to the emulated spindle. Gamma fusimotor drives were initially set to 0 Hz and activated to 80 Hz between the 3rd and 4th cycle. Again we observed distinctive spiking patterns in response to gamma static and dynamic stimuli.

### Population firing in response to various muscle stretches

**Figure 4:**
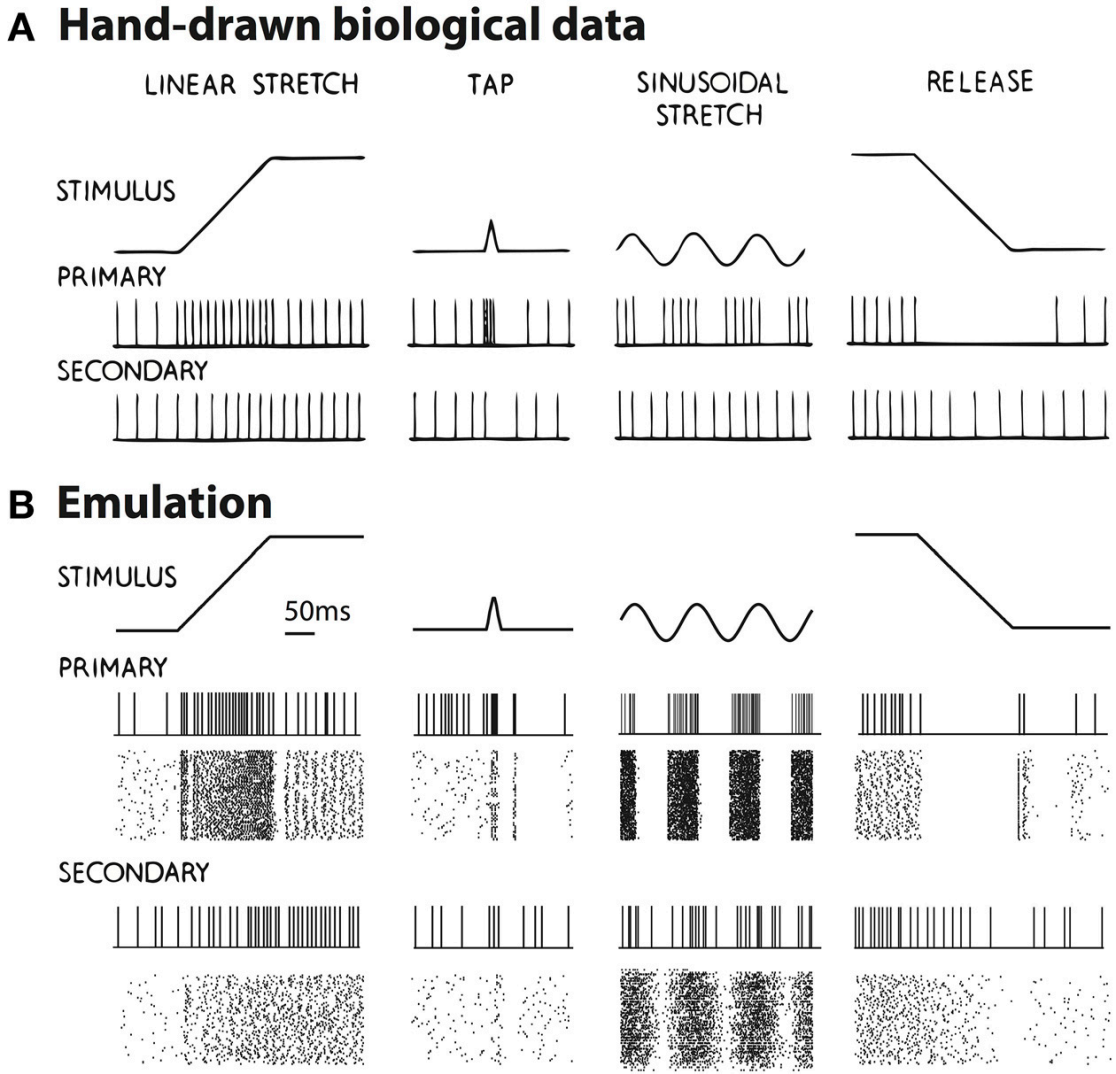
**(A)** The hand-drawn schematized response of muscle spindle summarized from experiments, in which the muscle withstood four types of stretching: linear stretch, tap, sinusoidal stretch, and release (Matthews, 1964). Reproduced with permission. **(B)** Emulated spikes from a representative neuron and the neuron ensemble when the four types of muscle stretching were applied. The raster shows 128 Primary afferent neurons and 128 Secondary afferent neurons. Changes in spike pattern are similar between experimental and emulated results, with emulated results showing irregularities in spiking due to added random noise. Note that in the case of muscle release (last column), the Primary afferents show a burst of firing after the release suddenly stopped. This burst was not schematized in **(A)**, probably because sudden stops are usually smoothed significantly by skin, ligaments, and other tissues.

One significant advantage of neuromorphic emulation is that the spikes of a large number of neurons can be stored and analyzed together with many other emulated activities. Here we test whether the neuron ensemble shows expected spiking behaviors in response to various types of stretching waveforms. Figure 4A shows four types of stretch waveforms (linear stretch, tap, sinusoidal stretch and release) along with hand-drawn schematized spike responses summarized from experimental observations (Matthews, 1964) in absence of fusimotor drive, reprinted with permission.

The emulated responses are shown in Figure 4B. In all cases except for muscle release, the emulated spike train from a sample neuron matches the empirical responses. In addition, both the Primary and Secondary ensembles, each comprising 128 neurons, show variable but congruent patterns compared to a single neuron. The only noticeable exception is in muscle release (Figure 4B, rightmost column), where the Primary afferents show a burst of firing after the release stops. This burst is caused by the fact that a sudden stop is equivalent to a momentary stretch at very high velocity. It was not schematized in Figure 4A, however, probably because the original figure was hand-drawn such that some details were unnecessarily omitted. According to Poliakov and Miles (1994), sudden bursts of electromyography (EMG) were indeed present at the end of masseter releasing. Such discrepancy demonstrated the ability of our platform to validate inconsistent observations.

In addition, there is a noticeable distinction between the regularity of spikes of Figures 4A,B. One explanation is that our emulation explicitly introduced synaptic noise, and therefore the individual neuron will exhibit a Poisson-like random firing pattern. This particular randomness was perhaps not the focus when Figure 4A was schematized.

**Figure 5:**
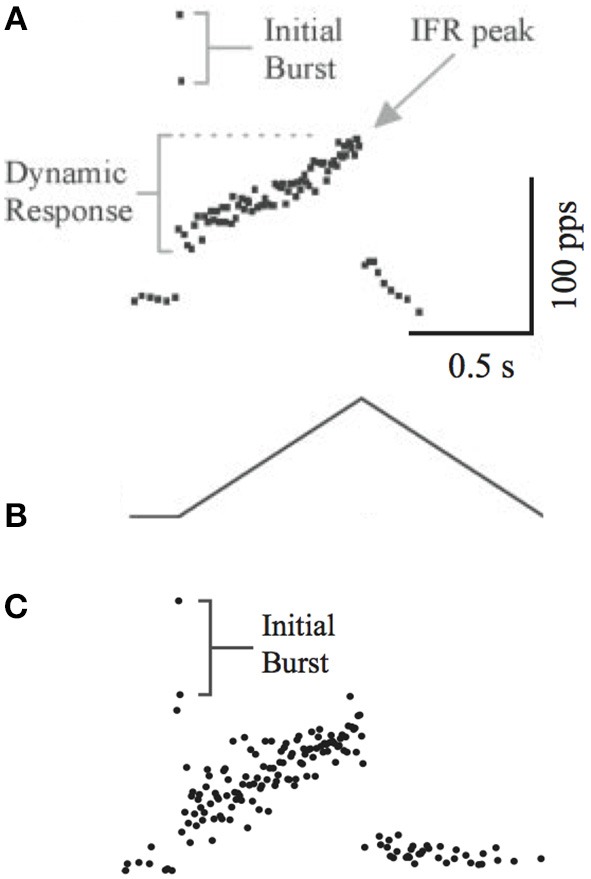
**(A)** The initial burst of instantaneous frequency of spindle afferent firing. **(B)** The triangular stretching waveform used by Haftel et al. (2004). **(A,B)** reproduced with permission. **(C)** The instantaneous frequency calculated from 20 trials of emulated stretch using the same waveform. The initial burst is evident.

Another feature of muscle spindle is that its afferents usually spike at a significantly high rate during the beginning of muscle stretch, resulting in an “initial burst.” This feature was measure in rats by Haftel et al. (2004) as shown in Figure 5A. We used the same triangular waveform (Figure 5B) to stretch our emulated spindle for 25 repetitions. The instantaneous frequency averaged across 25 repetitions (Figure 5C) showed similar initial bursts compared to experimental data.

### Correlation between rate-based model and spike-based emulation

**Figure 6:**
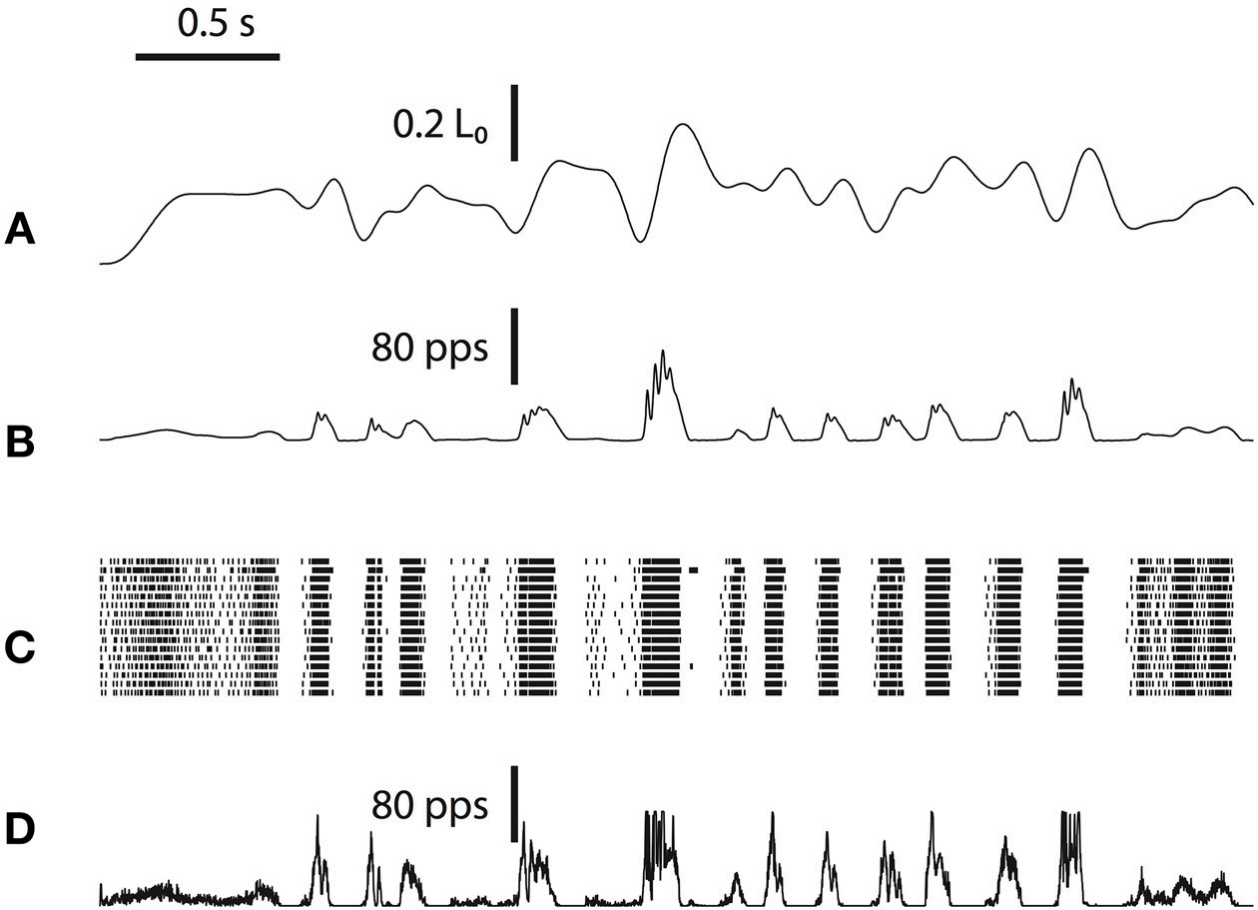
**White-noise stimulus to the emulated spindle and its spiking responses. (A)** A sample path of the low-pass filtered white-noise stretch waveform. **(B)** The Group Ia firing-rate produced by spindle model. **(C)** Group Ia afferent raster produced by neuromorphic emulation. **(D)** The spike count summarized from the spike raster. Similar white-noise analysis was performed for Group II afferents (not shown).

We tested the spindle afferents using a pseudo-white-noise waveform, which examines the input-output relationship of a dynamic system with rich frequency components (Marmarelis, 2004). The waveform includes a series of pseudorandom numbers low-pass filtered at 5 Hz cutoff frequency. The low-pass filtering screens out the unrealistic high-frequency stretching that is usually damped by skin and ligaments. One sample of a 4-s stretch is shown in Figure 6A. The corresponding Primary Ia response in firing rate (Figure 6B) generates stochastic spiking shown in a raster plot (Figure 6C, raster of 16 neurons). The firing rate produced by the original spindle model is compared to the total spike count recorded from the 256 emulated spindle afferent neurons (Figure 6D). The total spike count shows irregularity and variability in each run because of the stochastic spiking in the emulated neuron population.

We stretched the spindle for an equivalent of 160 s in real-time using the low-pass filtered pseudo-white-noise waveform. The total spike count is significantly correlated with the expected firing rate for both Primary Ia (*p* < 0.0001, *r* = 0.813) and Secondary II (*p* < 0.0001, *r* = 0.810). These results verify that our emulation provides a consistent spike-based representation of muscle length and lengthening velocity; the spiking outcome is statistically compatible with the original rate-based spindle model on which it is based.

### Comparison to human spindle afferents

**Figure 7:**
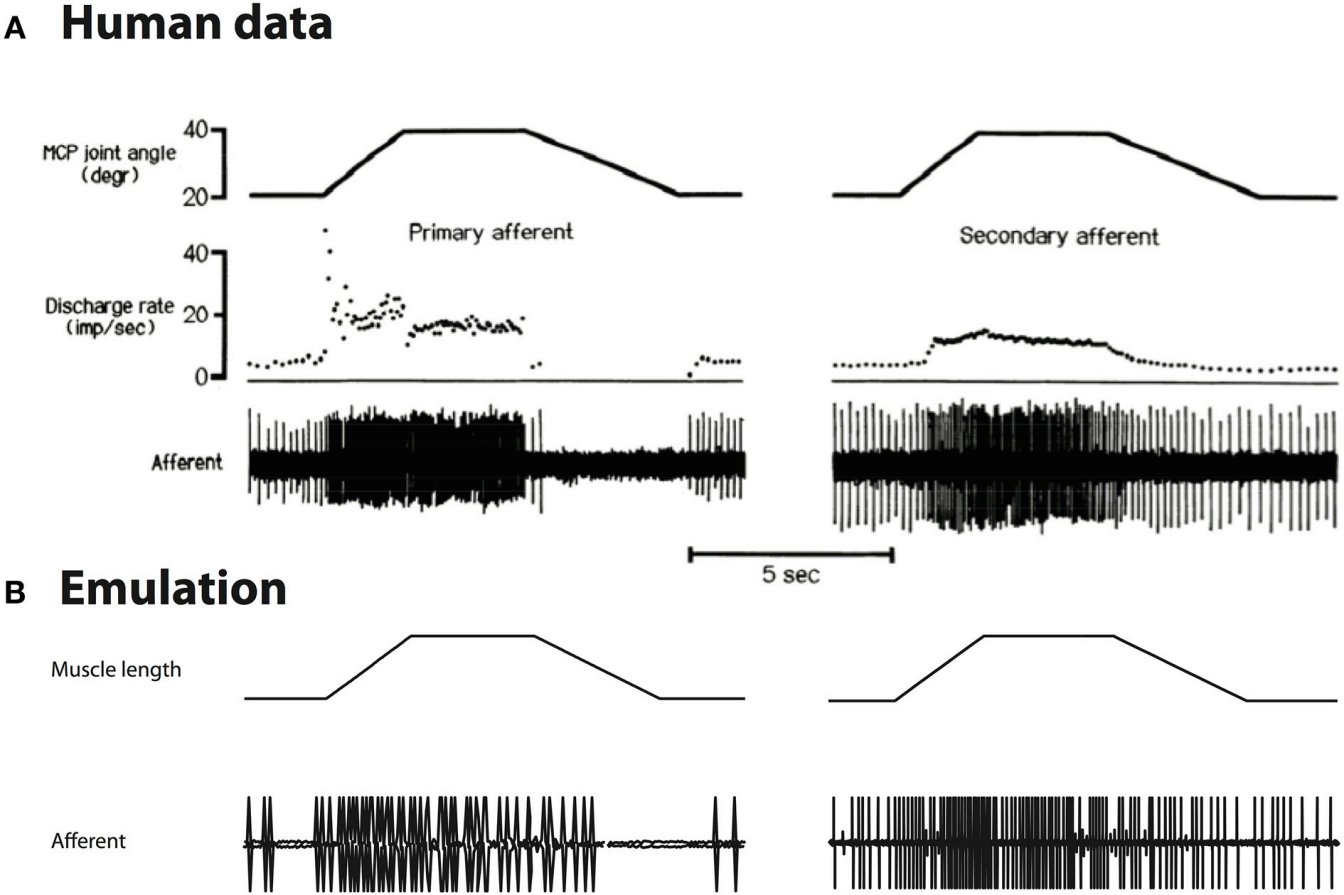
**(A)** Stretch responses from human spindles Edin and Vallbo (1990), reproduced with permission. **(B)** Emulated spindle responses using the same waveforms. The overall firing rate is lower than human data, and the contrast between different lengths of muscle is less prominent.

Although the original spindle model was developed based on cat soleus spindles, we replicated the experiments done with human spindle to compare our emulation with human data. We introduced 0.7*L*_0_ stretch using the waveform reported by Edin and Vallbo (1990). The human spindle recording and emulated activity are shown in Figure 7.

As can be seen, the overall spike rate is lower in emulation than human data; the emulated spike rates show less contrast between long and short muscle length as compared to human data, i.e., reduced static response. These differences are compatible with the finding that human spindles contain more intrafusal fibers, which are probably dominated by nuclear chain (static) fiber (reviewed in Prochazka and Hulliger, 1983). Quantitative comparison is not performed due to limited human data. In principle, the spindle model could be tuned to fit human spindles by manipulating the damping terms of the spindle fiber.

## Discussion

Using our recently developed technique of neuromorphic emulation on programmable digital VLSI hardware, we showed that a collection of spiking afferent fibers driven by a detailed model of muscle spindles suffice to produce biologically-realistic spindle afferents. The observed firing pattern from emulated spindle neurons matches classical intramuscular recordings from cat soleus muscle, and the emulated responses to gamma fusimotor drive show no qualitative difference from experimental recordings. All emulations can be accomplished at 365× real-time, which allows estimating long-term changes and large population behavior efficiently. The emulated spindles differ from human spindle activity but the difference is compatible with the known difference between cat and human spindles. Our results provide a strong validation of using neuromorphic emulation as a testbed for neurophysiological studies. We can now test the roles of more complex structures, such as realistic muscles, inhibitory neurons, or supra-spinal circuitry in producing movement behavior. The multi-scale design enables us to emulate pathological conditions that are physiologically tenable, e.g., death of neurons or absence of gamma fusimotor drive. It creates an essential step toward investigating how such pathological conditions could contribute to disease progression in childhood.

Synthetic emulation using neuromorphic hardware provides three major advantages compared to empirically studying the real biological system. First, it isolates the subsystem-of-interest from the compounding factors that are very difficult to tease out *in-vivo*, including studying the spinal reflex isolated from supra-spinal influence. Second, it allows interventions that are usually difficult to introduce when studying biological systems, e.g., differentially adjusting the relative weight of gamma dynamic vs. static drive. Third, the hardware acceleration allows for predicting the system's long-term development with significantly less time. Our vision for the synthetic approach is not to replace empirical studies but rather to inspire testable hypotheses for experiments and clinical applications. Another important feature is that this platform can be used to verify the source of motor variability from different physiological origins, such as the intrinsic firing variability of neurons, motor noise associated with muscles, or the individual properties of mechanoreceptors.

The purpose of this study is to validate the neuromorphic hardware as a testbed for spindle activity, therefore we focused on (1) implementing a selected model of spindle (Mileusnic et al., 2006), (2) adapting it to enable spiking afferents, (3) accelerating it to 365× real-time; we did not focus on improving the model to match more experimental data than it originally could. Nevertheless, the limitations of this spindle model should be acknowledged. One major limitation is that the long-known thixotropic property of spindle activity (Hasan and Houk, 1975) was not captured; also the model was based on cat data, thus it must be re-calibrated for modeling human movement disorders. It is worth noting that other excellent models of spindle also exist (e.g., Hasan, 1983; Lin and Crago, 2002), they either used a less computationally expensive approach to model the non-linear velocity dependency (Hasan, 1983), or succeeded in unifying spindle and Golgi Tendon Organ with the same structure (Lin and Crago, 2002). Recent work also refined the original spindle model by examining the non-linearity in its components (Lan and He, 2012). Our design of the platform is open and flexible for including additional features in future improvements, or switching to different models if necessary.

The original model of spindle (Mileusnic et al., 2006) focused on the firing rates of spindle afferents instead of their spiking patterns. Such rate-based models are incompatible with our overall goal of disease emulation, where the spike timing is crucial for motor variability and neuroplasticity. In acknowledgement of the soundness of the original rate-based spindle model, our major improvement is to enable a large number of spiking neurons driven by a spindle. Moreover, the original model was developed in Matlab Simulink operating slower than real-time, while our hardware implementation permits faster-than-real-time performance. This is the first physical, portable, and realistic proprioceptor that can provide synthetic proprioception for robots, virtual neurophysiological studies, and prediction of clinical outcomes.

### Conflict of interest statement

The authors declare that the research was conducted in the absence of any commercial or financial relationships that could be construed as a potential conflict of interest.
